# Ground truth and demographic data for ultrasound assessment of thoracolumbar fascia deformation and shearing

**DOI:** 10.1038/s41597-025-05701-6

**Published:** 2025-08-11

**Authors:** Andreas Brandl, Robbert van Amstel, Robert Schleip

**Affiliations:** 1 Conservative and Rehabilitative Orthopedics, TUM School of Medicine and Health, Technical University of Munich, Munich, Germany; 2 Department for Medical Professions, Diploma Hochschule, Bad Sooden-Allendorf, Germany; 3 Department of Human Movement Sciences, Faculty of Behavioural and Movement Sciences, Amsterdam Movement Sciences, Vrije Universiteit Amsterdam, Amsterdam, Netherlands

**Keywords:** Risk factors, Medical research

## Abstract

Restricted mobility and/or deformability of the thoracolumbar fascia (TLF) could be a diagnostic criterion or a risk factor for the development of low back pain. Therefore, ultrasound measurement techniques to quantify TLF characteristics have been used for over two decades without comprehensive validation. The aim of this paper is to present data to validate ultrasound methods for the assessment of shear, sliding motion and deformation based on distance measurement of anatomical landmarks or speckle tracking. In addition, epidemiological data with deformation values of the TLF are offered. A sliding device with polyurethane tissue phantoms was developed to generate ground truth values for different distances and velocities. The resulting dataset includes 36 ultrasound videos for typical TLF sliding distances of 3–20 mm, 10 videos for various velocities between 2.4 and 5.4 mm/s, demographic data on TLF deformation from 199 individuals, and a MATLAB example script for use with image processing software. For the first time, the dataset provides researchers with ground truth values to validate their ultrasound methods.

## Background & Summary

Low back pain (LBP) is the leading cause of years lived with disability worldwide^1^, and the expense of it is putting significant economic pressure on healthcare systems^2^. With only a third of LBP patients recovering after three months and 33.3% recurrent episodes often occurring within the next year^3^, LBP has become a compelling global problem^4^.

Despite of decades of research often there is no linear cause effect between specific conditions like disc disease or radiculopathy and LBP^5–8^. Nevertheless, certain predictors have emerged, such as older age, obesity, and physical exertion. Particularly, acute LBP episodes and psychosocial health issues are significant risk factors for LBP progression^5,9^.

There is increasing evidence that another risk factor is the limited mobility and/or deformation of the thoracolumbar fascia (TLF)^10–13^, which is a multilayered aponeurotic structure that envelops the erector spinae muscles^14^. Static and dynamic real-time ultrasound with subsequent analysis of ultrasound images and videos were used to assess shear capacity, gliding mobility, and deformation. These techniques are frequently used in research to gain a better insight into the kinematics of the TLF.

However, since the first TLF shear measurement method was introduced 23 years ago^10^, no comprehensive validation and reliability assessment has been performed apart from intra-study intrarater tests with very small samples. Therefore, as a first step, we conducted a reliability study for a static measurement method for TLF deformation and its ability to differentiate LBP patients from healthy individuals^15^. Nevertheless, Mohr *et al*.^16^ demonstrated that such a static approach using external references may overestimate the actual values of the deformation measurements. Dynamic ultrasound assessments, on the other hand, could be post-processed by video analysis with speckle tracking, which is also frequently used in research, although it is not even validated^10,17–20^. Speckle tracking analysis uses the unique grayscale-encoded patterns of pixels in a video frame image, which result from the interference of scattered ultrasound waves from small tissue structures, allowing for the analysis of tissue movement and deformation over time^21–23^. Combining the lack of validity with the recently discovered potential benefits of ultrasonography in the context of LBP monitoring, the urgent need for validation becomes apparent.

In this data descriptor, we present a dataset with ground truth values for the validation of ultrasound shear, sliding motion and deformation assessment methods based on distance measurement of anatomical landmarks or speckle tracking. In addition, we offer epidemiologic data with deformation data of the TLF (TLFD) in healthy individuals and LBP patients, which will be continuously expanded with new data in the following years. The data set is published in the open Zenodo repository^24^.

## Methods

### Generation of ground truth data

**Fig. 1:**
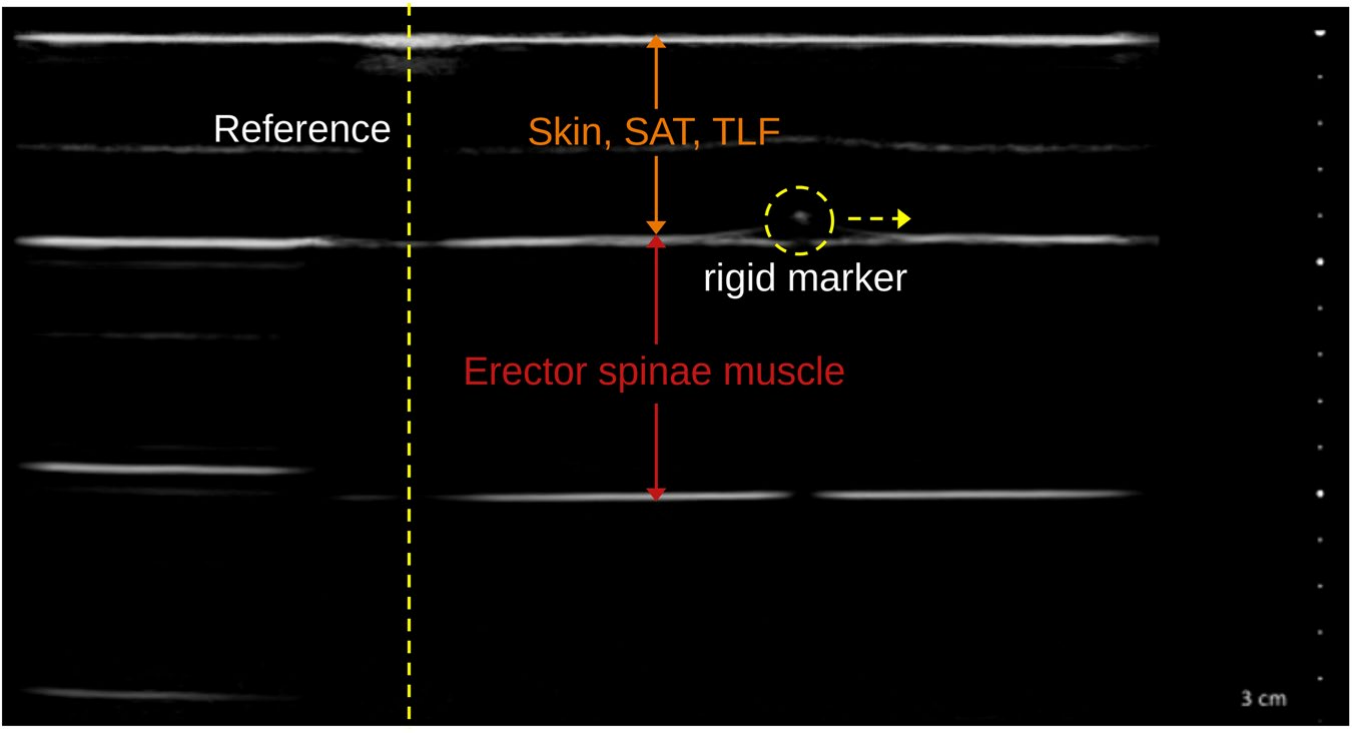
Ultrasound image of the phantom tissue model. Skin, SAT, TLF: gel pad mimics the cutis, subcutaneous adipose tissue and the thoracolumbar fascia (1^st^ layer; fixed attached). The 2^nd^ layer mimics the erector spinae muscle with the lateral displaced rigid marker (yellow dashed circle) attached on the top of the 2^nd^ layer (freely movable). The reference created by the nylon thread produces a vertical shadow in the ultrasound image (yellow dashed line).

The ultrasound images were acquired using a computer-controlled linear actuator that sheared two gel pad tissue phantoms against each other. To create an artificial shadow as a reference marker in the ultrasound image, a nylon thread was placed between the first gel pad and the probe^16^. In addition, a high-density, 0.5 mm thick reflective polyethylene marker was attached to the second gel pad to serve as a rigid marker^25,26^. The design of the marker was based on the morphological description of the junction between the latissimus dorsi muscle and the thoracolumbar fascia by Willard *et al*.^14^, which has been used as an ultrasound reference in several previous studies^8,15^ (Fig. 1). The displacement of the rigid marker on the second gel pad was tracked with dynamic ultrasound imaging (Clarius L15 HD3 linear transducer, 15 MHz; Clarius Mobile Health Corp., Canada). The typical TLFD (3–20 mm) in dynamic ultrasonic measurements and their velocities (2.5–5 mm/s) were investigated based on previous studies^27,28^. The video resolution for post-processing was 800 × 800 pixels with a frame rate of 3 frames per second. This generates video files that can also be processed with less powerful image processing software (approx. 4 MB memory for each file). However, this could limit the usability with regard to the validation of methods for the detection of fast tissue movements.

### Subject characteristics of epidemiological data

The epidemiological data is a collection of retrospectively collected TLFD values from healthy participants and patients with acute LBP. The data originate from various studies or investigations. As usual for musculoskeletal ultrasound examinations, linear transducers from different manufacturers with 10 to 15 MHz resolution were used (the specific type is indicated for each measurement). The DOI number, if available, of the respective study is given for better transparency. The dataset currently comprises a total of 199 people, 97 men, 102 women, 44 patients with LBP and 155 healthy people^24^. It is planned to continuously expand the epidemiological data with new diagnostic data from multicentre sources in order to obtain a comprehensive database with reference values for TLFD under different conditions (e.g. gender, age, BMI, health status). The epidemiological data acquisition policies has been reviewed and approved by the ethical committee of the Diploma Hochschule, Germany (Nr.1014/2021), has been carried out in accordance with the declaration of Helsinki and has obtained written informed consent to participation and data sharing from the participants^29^. The data was completely anonymized in accordance with the German General Data Protection Regulation, i.e. neither the authors nor users of the dataset^24^ can identify the participants^30^. Therefore, the original data with the participant information was imported into R (v4.1.2; R Core Team 2021)^31^ and then anonymized using the “anonymizer” package (version 0.2.0)^32^ and transcribed into an OpenDocument table.

### Image acquisition for epidemiological data

Ultrasound measurement of the TLFD was originally described by Brandl *et al*. in a series of publications^27,28,33^. The method uses the junction between the latissimus dorsi and the TLF as an anatomical landmark. A full description of the protocol can be found at 10.17504/protocols.io.eq.2lyjbmwlx9/v1.

Here is a brief summary: Participants sit on a treatment table and bend their trunk under the control of an examiner until a flexion angle of about 60 degrees is reached (starting position). A static US image is then taken with a high-resolution (>12 MHz) ultrasound linear transducer. The participants then extend their trunk to an angle of 0 degrees (ending position) and another static ultrasound image is acquired. The distance between the junction of the latissimus dorsi muscle and the TLF and an artificial reference marked by a reflective tape on the skin is measured. The relative change in the distance between the starting and ending positions represents the extent of the TLFD.

This method showed excellent intra-rater reliability (ICC: .92) and good inter-rater reliability (ICC: .78)^33^, and validity also proved to be excellent on the basis of this dataset (ICC: .99)^34^.

## Data Records

The full dataset is available at 10.5281/zenodo.15606741 in ZIP compressed format^24^. This includes five folders with demographic data, 36 videos with ground truth data for distances between 3 and 20 mm, 10 videos with ground truth data for speeds from 2.41 mm/s to 5.36 mm/s and one MATLAB script to demonstrate use with speckle tracking. All demographic or ground truth values are documented in OpenDocument spreadsheets (*.ods) in the respective folder. The dataset also includes a video demonstrating how the customized device works, an additional full description and a step-by-step validation protocol to facilitate the application of the ground truth data in the root directory. Table 1 shows the structure of the dataset.

**Table 1:** Structure of the dataset.

Folder	ODS Table/File	Sheets	Description
	Device setup.mp4		Video demonstration of the device
	README.md		Text file with dataset description
TLFD_demographics	TLFD_demographics	Demographics	Contains the data
		Abbreviations	Abbreviations and units used in the table
TLFD_GT\TLFD_GT_distances	D_TLFD_*.avi		Ground truth videos distances measurements
	TLFD_GT_distances	Distances	Contains ground truth distance data and filenames of ultrasound
		Abbreviations	Abbreviations and units used in the table
TLFD_GT\TLFD_GT_speeds	S_TLFD_GT_*_RPM_*.mp4		Ground truth videos speed measurements
	TLFD_GT_speeds	Speeds	Contains ground truth speed data and filenames of ultrasound
		Abbreviations	Abbreviations and units used in the table
MATLAB			Complete demo files for speckle tracking analysis with Kinovea
MATLAB\Readme			Detailed instructions for using the MATLAB script

## Technical Validation

### Gel pad tissue phantoms

A two-layer phantom tissue model was used to standardize the ultrasound measurements of lateral displacement in rigid kinematics. This model is based on previously developed polyurethane gel pads (21 cm × 31 cm; Technogel Germany GmbH, Berlingrode) that simulate the lumbar tissue laterally at the level of L3. For a detailed description, see Bartsch *et al*.^35^. The model has been used in previous reliability studies for devices evaluating myofascial tissues in different layered structures^35–37^. The two-layer model was used to capture ground truth, with the first layer representing the cutis, subcutaneous adipose tissue and TLF. The second layer represented the erector spinae muscle. The morphological characteristics in terms of thickness and stiffness were determined from a literature search, resulting in a thickness of 10 mm for both layers and corresponding to the upper limit for reliable ultrasound evaluation^35^.

Although the use of gel pad phantoms to mimic living tissue has proven to be reliable and easy to use^35^, human biological material also has additional structures such as blood and nerve vessels, fat and fluid infiltrations, which may affect stiffness and shear properties. Therefore, the use of phantoms in this approach is a technically necessary simplification compared to biological material.

### Tissue sliding device

**Fig. 2:**
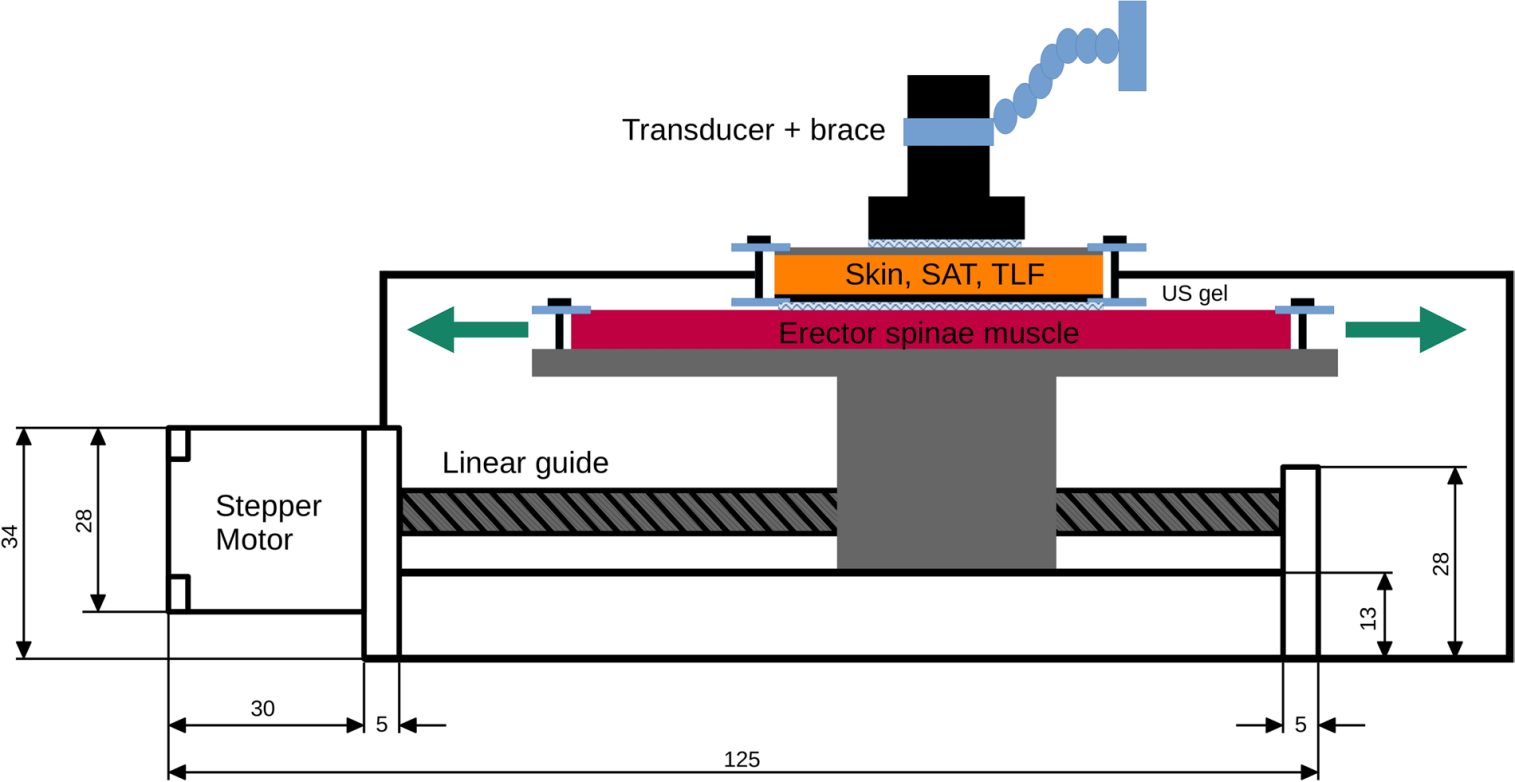
Tissue sliding device. Skin, SAT, TLF: gel pad mimics the cutis, subcutaneous adipose tissue and the thoracolumbar fascia (1^st^ layer; fixed attached). The 2^nd^ layer mimics the erector spinae muscle (freely movable). The transducer is vertically adjusted with a spirit level and fixed installed with a brace. The transducer and the two layers are separated by ultrasound gel.

We have developed a customized sliding device for tissue in which the second gel pad is attached to a platform mounted on a linear actuator (SFU1605-100 mm, Shandong Sair Mechanical Guide Co. LTD, Gaotang, China) driven by a stepper motor (NEMA17, ACT Motor GmbH, Bremen, Germany). With this device, the second gel pad can be slid sideways under the first gel pad (Fig. 2).

**Fig. 3:**
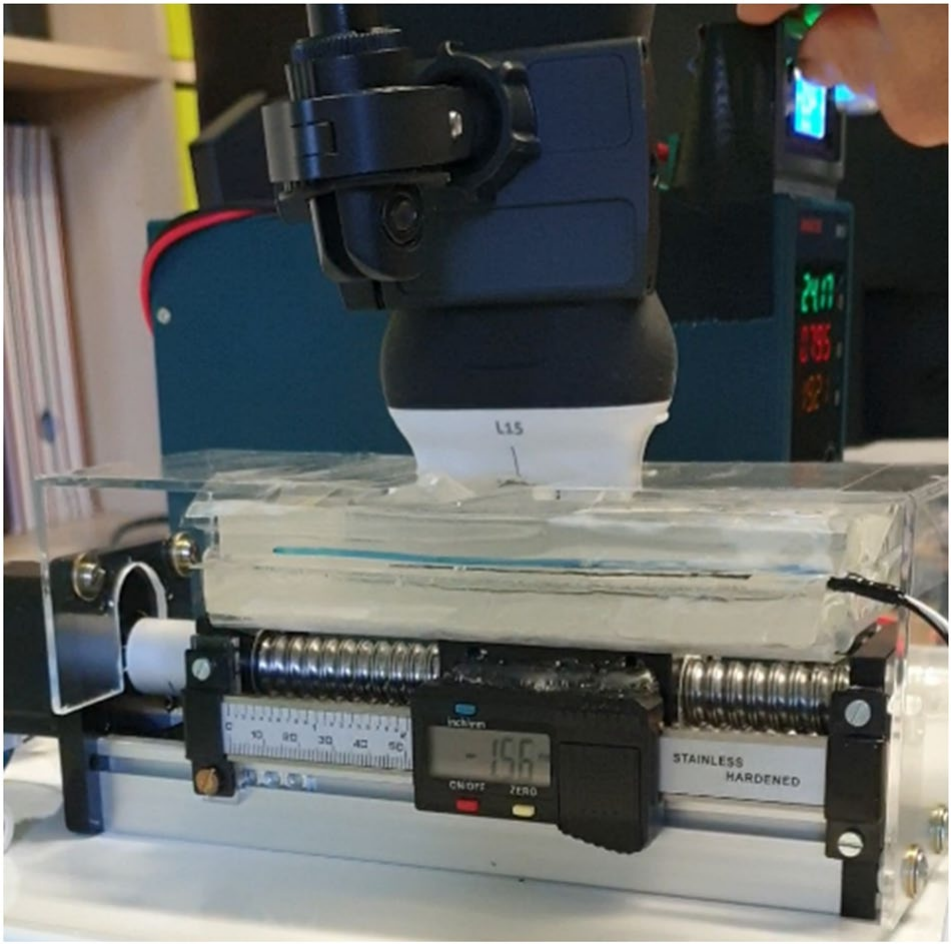
Tissue sliding device. The top gel pad is fixed while the bottom layer is free to move. The transducer is vertically aligned with a spirit level and firmly installed with a clamp. The transducer and the two layers are separated by ultrasonic gel. The mounted digital caliper measures ground truth with an accuracy of ±2 µm.

Ultrasound gel (DocCheck Ultrasonic Gel, Gello GmbH Geltechnik, Ahaus-Wüllen, Germany) was used between the gel pads to ensure ultrasonic transmission, reduce friction and enable smooth sliding. A strain gauge sensor (ZD10-100, Shenzhen Lanqi Technology Co. LTD, Shenzhen, China) was attached to the layers to detect any shear deformation during their relative movement. The stepper motor, which was equipped with a digital caliper, kept the previously set speeds constant with a positioning accuracy of ±1 µm. This ensured that the displacement of the first gel pad relative to the second was reliably documented and could be used as the basis for the measurement. No shear deformations were detected within the gel pads (<0.1 mm). The measurement uncertainty for the distance was ±2 µm, k = 2 (95% confidence interval) according to the “Guide to the Expression of Uncertainty in Measurement” (GUM; ISO 1993). By using this standardized model, the accuracy and reliability of the analysis of tissue displacement has been improved (Fig. 3; Device setup.mp4).

## Usage Notes

Here, we demonstrate the use of ground truth data^24^ with Kinovea, an open source image processing software^38^. The tracked speckle data from the respective ground truth video (e.g. D_TLFD_01.avi) were analyzed using a custom MATLAB script (The MathWorks, Natick, MA, USA). Full tissue displacement was calculated with the Eq. (1):

1 $${{Lateral}}_{{displacement}}{\int }_{0}^{10}\triangle {{distance}}_{({Pxi}-S)}\left(t\right){dt}$$

Lateral displacement (Pxi) was measured from the starting position (S) to the ending position over 10 seconds. The analysis began by determining a conversion factor between pixels and millimeters using the scale bar in the ultrasound image and calibration. This factor converted displacement data to millimeters. Next, the time frame was established based on the number of frames and frame rate. The stepper motor of the tissue sliding device was digitally controlled at a constant speed, with ground truth data obtained from initial displacement and speed, enabling recalculation of displacement over time for comparison with ultrasound tracking data. Data normalization was done for time shifts and phases: starting position, sliding motion, and ending position for both ground truth and ultrasound speckle tracking analysis. Ultrasound data was processed using a smoothing function. Additional observations included calculating spatial tracking error, creating histograms of tracking errors, and generating scatter plots and time series plots to visualize the data and its spatial measurement error.

A recent study using this approach to validate speckle tracking analysis for the assessment of TLF sliding mobility showed excellent test-retest reliability (ICC: .95) and very high validity against this ground-truth data (r = .97)^39^.

The example MATLAB script presented here is available in the “MATLAB” folder in the dataset.

## Data Availability

No programming code was used in the construction of the dataset.
